# A new species of *Hessebius* Verhoeff, 1941 (Lithobiomorpha, Lithobiidae) from China with a key to species

**DOI:** 10.3897/BDJ.9.e72336

**Published:** 2021-08-18

**Authors:** Sujian Pei, Huiqin Ma, Yanmin Lu, Haipeng Liu, Kuijing Liang

**Affiliations:** 1 Institute of Myriapodology, School of Life Sciences, Hengshui University, Hebei 053000, Hengshui, China Institute of Myriapodology, School of Life Sciences, Hengshui University, Hebei 053000 Hengshui China; 2 Hebei Key Laboratory of Wetland Ecology and Conservation, Hebei 053000, Hengshui, China Hebei Key Laboratory of Wetland Ecology and Conservation, Hebei 053000 Hengshui China

**Keywords:** Lithobiomorpha, *
Hessebius
*, new species, Hengshui Lake National Nature Reserve, Hebei Province, China

## Abstract

**Background:**

The myriapod fauna of China is still poorly known and very little attention had been paid to the study of Lithobiomorpha, with only 100 species/subspecies hitherto known from the country, among which are only seven species of *Hessebius*. Here we are describing a new species of the genus *Hessebius*.

**New information:**

A new lithobiid species, *Hessebiuscrassifemoralis* sp. nov., is described and illustrated from Hengshui Lake National Nature Reserve, Hebei Province, China. The new species is compared with *H.luquensis* Qiao, Qin, Ma, Su & Zhang, 2018 from Gansu Province, China. A key to Chinese species, based on adult specimens, is provided. Type specimens and other material are deposited in the School of Life Sciences, Hengshui University, Hengshui, China.

## Introduction

*Hessebius* was originally proposed as a genus in the family Lithobiidae by Verhoeff (1941) to accommodate the species *H.kosswigi* Verhoeff, 1941 and *H.tauricus* Verhoeff, 1941 described from Turkey. The latter species was re-assigned to the genus *Lithobius* Leach, 1814 by Zapparoli (1999). Zalesskaja (1978), Eason (1981), Pei et al. (2010) and Zapparoli and Edgecombe (2011) had debated the taxonomic status of *Hessebius* and considered it as having generic rank and selected *H.kosswigi* Verhoeff, 1941 from Turkey, by subsequent designation, as type species (Verhoeff 1941, Zalesskaja 1978, Eason 1981). The genus is characterised by the following traits: antennae of 17–23 articles, usually 20, 4–15 ocelli, forcipular coxosternal teeth 2+2; tergites without posterior triangular projections; legs 14 and 15 thicker than the anterior ones in female, both thicker in male; coxal pores 4–7; first article of female gonopods with 2+2 spurs, second article of female gonopods with a massive expansion and projection on the dorsolateral ridge and a long claw, sometimes with a stout lateral tooth at its base. The recorded distribution of the genus *Hessebius* extends from Mongolia and south-east China through central Asia (Kazakhstan, Kyrgyzstan, Tajikistan, Turkmenistan), southern Urals, south-west Russia (Kalmykia and adjacent areas), westwards up to the Middle East and eastern Mediterranean basin (Iran, Armenia, south-west Turkey, Rhodes, Cyprus, Syria, Palestine, Israel, Jordan, north Egypt, Cyrenaica) (Pei et al. 2010). Presently, the genus *Hessebius* comprises 16 species (Bonato et al. 2016), amongst which are only seven species from China. The present study deals with the description of a new species of *Hessebius* from Hebei Province, China. A key to the known Chinese species of the genus, based on adult specimens, is presented.

## Materials and methods

Specimens were collected under leaf litter or stones and preserved in 75% ethanol. Illustrations and measurements were produced using a ZEISS SteREO Discovery.V20 microscope equipped with an Abbe drawing tube and an ocular micrometre and Axiocam 512 colour. The colour description is based on specimens fixed in 75% ethanol. The body length is measured from the anterior margin of the cephalic plate to the posterior end of the postpedal tergite. The terminology of the external anatomy follows Bonato et al. (2010). The type specimens and other material examined are deposited in the School of Life Sciences, Hengshui University, Hengshui, China (HUSLS). The following abbreviations are used in the text and in the tables: a – anterior; C – coxa; F – femur; m – median; P – prefemur; p – posterior; S, SS – sternite, sternites; T, TT – tergite, tergites; Ti – tibia; Tr – trochanter; Ta – tarsus.

## Taxon treatments

### Hessebius crassifemoralis

Pei, Ma, Lu, Liu & Liang, 2021 sp. n.

6052AC1A-AB01-5DF2-945C-D73F732AC739

293BB0D6-D9DA-4971-8017-430612B8B6C0

#### Materials

- **Type status:**Holotype. **Occurrence:** recordedBy: Pei, Ma, Lu, Liu & Liang; individualCount: 1; sex: male; lifeStage: adult; **Taxon:** scientificName: *Hessebiuscrassifemoralis*; kingdom: Animalia; phylum: Arthropoda; class: Chilopoda; order: Lithobiomorpha; family: Lithobiidae; genus: Hessebius; taxonRank: species; taxonomicStatus: species; **Location:** continent: Asia; country: China; stateProvince: Hebei; county: Taocheng; locality: Longyuan hotel, Hebei Hengshui Lake National Nature Reserve, Hebei Province, China; verbatimElevation: 24 m a.s.l.; decimalLatitude: 37.649011; decimalLongitude: 115.659337; **Identification:** identifiedBy: Huiqin Ma; dateIdentified: 2020; **Event:** eventDate: 24/07/2020; **Record Level:** collectionCode: Myriapoda; basisOfRecord: PreservedSpecimen
- **Type status:**Paratype. **Occurrence:** recordedBy: Pei, Ma, Lu, Liu & Liang; individualCount: 7; sex: 6 male and 1 female; lifeStage: adult; **Taxon:** scientificName: *Hessebiuscrassifemoralis*; kingdom: Animalia; phylum: Arthropoda; class: Chilopoda; order: Lithobiomorpha; family: Lithobiidae; genus: Hessebius; taxonRank: species; taxonomicStatus: species; **Location:** continent: Asia; country: China; stateProvince: Hebei; county: Taocheng; locality: Longyuan hotel, Hebei Hengshui Lake National Nature Reserve, Hebei Province, China; verbatimElevation: 24 m a.s.l.; decimalLatitude: 37.649011; decimalLongitude: 115.659337; **Identification:** identifiedBy: Huiqin Ma; dateIdentified: 2020; **Event:** eventDate: 24/07/2020; **Record Level:** collectionCode: Myriapoda; basisOfRecord: PreservedSpecimen
- **Type status:**Other material. **Occurrence:** recordedBy: Pei, Ma, Lu, Liu & Liang; lifeStage: adult; **Taxon:** scientificName: *Hessebiuscrassifemoralis*; kingdom: Animalia; phylum: Arthropoda; class: Chilopoda; order: Lithobiomorpha; family: Lithobiidae; genus: Hessebius; taxonRank: species; taxonomicStatus: species; **Location:** continent: Asia; country: China; stateProvince: Hebei; county: Jizhou; locality: Weitun town, Jizhou County, Hengshui City, Hebei Province, China; verbatimElevation: 23 m a.s.l.; decimalLatitude: 37.608275; decimalLongitude: 115.640952; **Identification:** identifiedBy: Huiqin Ma; dateIdentified: 2020; **Event:** eventDate: 21/04/2013; **Record Level:** collectionCode: Myriapoda; basisOfRecord: PreservedSpecimen

#### Description

**Figure 1.**
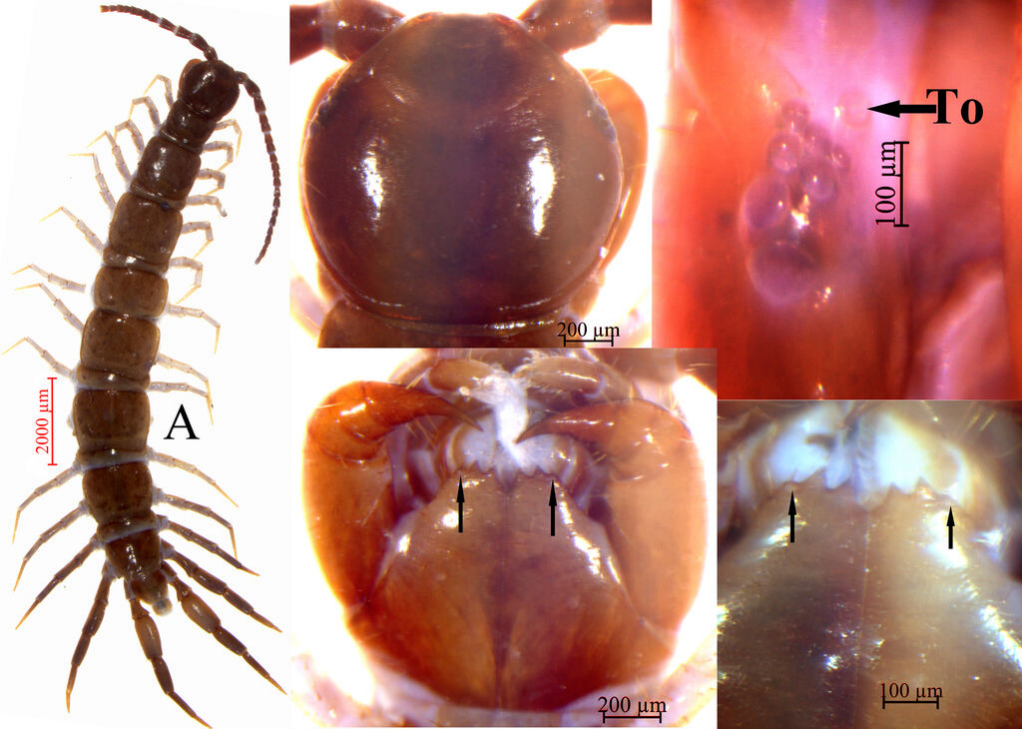
*Hessebiuscrassifemoralis* sp. nov. **A.** habitus, dorsal view, male holotype; **B.** cephalic plate, dorsal view, male holotype; **C.** ocelli and Tömösváry’s organ (To), lateral view, female paratype; **D.** cephalic plate, ventral view, male holotype; **E.** forcipular coxosternite, ventral view, male holotype.

Body (Fig. 1A). 9.9–15.2 mm long, cephalic plate 1.1–1.4 mm long, 1.2–1.4 mm wide.

Colour. Antennae chestnut-brown, the base nodes a deeper shade, the chestnut-brown gradually becomes yellow-brown at the end of articles 3–5, terminal article yellow brown; tergites yellow-brown with brownish hue, cephalic plate, TT13–15 deep red-brown; pleural region pale grey with pale purple hue; sternites pale yellow-brown; distal part of forcipules dark brown; basal and proximal parts of forcipules and forcipular coxosternite and SS14 and 15 yellow-brown; all legs pale yellow-brown with pale blackish hue; tarsus 1 of tibia yellow-brown, tarsus 2 more yellow on all legs.

Antennae. 18–23 articles, but antennae were damaged in the most part of the specimens examined and only eleven appear complete, usually 19–21 articles. Length of first antennal article slightly longer than width of the base, length of the remaining articles obviously larger than wide, the distalmost articles still significantly longer than wide, 2.3–3.1 times as long as wide; abundant setae on the antennal surface, fewer on the basal articles, gradual increasing in density to approximately the fifth article, then more or less constant.

Cephalic plate. Smooth, convex, slightly wider than long; tiny setae emerging from pores scattered very sparsely over the whole surface; frontal marginal ridge with shallow anterior median furrow; short to long setae scattered along the marginal ridge of the cephalic plate; lateral marginal ridge discontinuous, posterior margin continuous, almost straight, evidently wider than lateral marginal ridge (Fig. 1B).

Ocelli. Six to seven (commonly seven) oval to rounded ocelli on each side, from small to large, arranged in three irregular rows, the posterior ocellus the largest. Ventral ocelli slightly smaller than the dorsal, domed, translucent and usually darkly pigmented (Fig. 1C).

Tömösváry’s organ (Fig. 1C). Close to the ocelli, situated at anterolateral margin of the cephalic plate, the surrounding sclerotised area narrow, moderately smaller than the adjoining ocelli.

Coxosternite. Subtrapezoidal (Fig. 1D), anterior margin narrow, lateral margins slightly longer than medial margins; median diastema moderately deep, a narrow V-shape; anterior margin with 2+2 acute triangular teeth; porodonts slightly thicker, just posterolateral and separated from the lateral tooth, with slight bulge at base (Fig. 1D, E); long scattered setae on the ventral side of coxosternite, longer setae near the dental margin.

Tergites. Smooth, without wrinkles, dorsum slightly convex; short to long tiny setae emerging from pores scattered sparsely over the entire surface, near the margin with few long setae; TT1 and 3 narrower than the cephalic plate, T3 wider than the T1. T1 narrower postero-laterally than antero-laterally, generally inverted trapezoidal; lateral marginal ridges of all tergites continuous. Posterior margin of TT1, 3 and 5 continuous, posterior margin of TT8, 10, 12 and 14 discontinuous; posterior margin of TT1 and 3 straight, posterior marginal ridge of TT3 and 5 slightly concave, TT8, 10, 12 and 14 concave (Fig. 1A). Posterior angles of all tergites rounded, without triangular projections. From short to long, but miniscule setae scattered very sparsely over the surface.

Sternites. Posterior side of sternites narrower than anterior, generally inverted trapezoidal, smooth; setae emerging from sparsely scattered pores on the surface and lateral margin, very few short setae scattered sparsely among them; two pairs of approximately symmetrically arranged long setae on middle parts of anterior part of each sternite; with 2–5 very long setae in the anterior angles and with 1–3 very long setae in the posterior angles.

**Figure 2.**
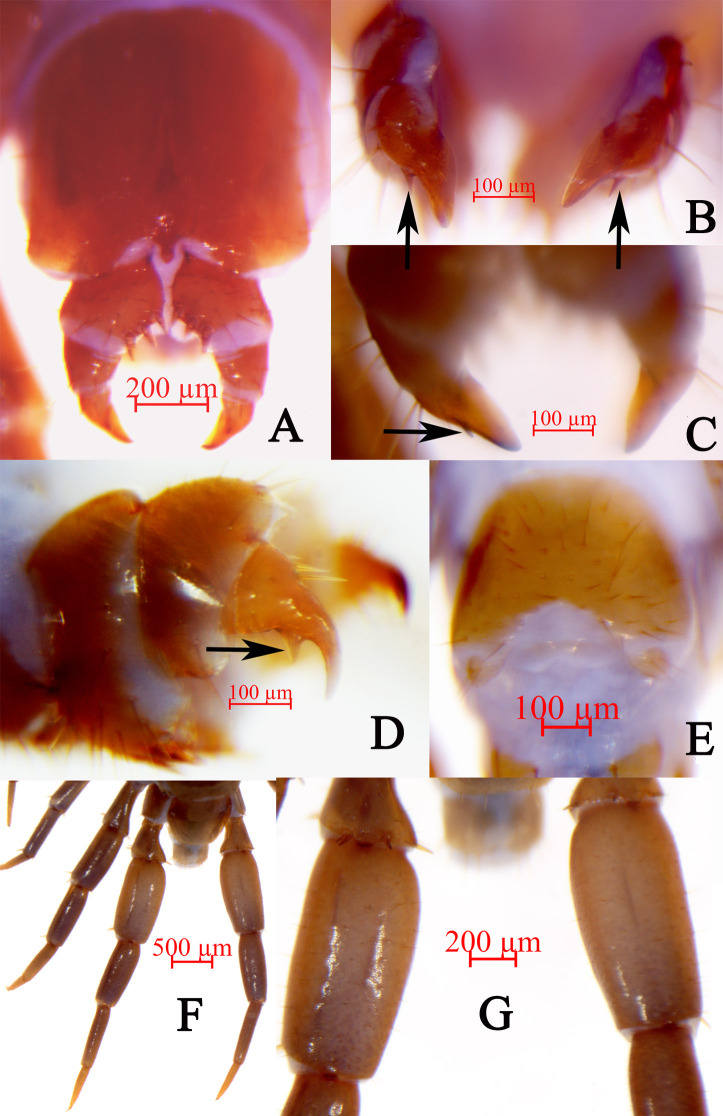
*Hessebiuscrassifemoralis* sp. nov. **A.** posterior segments and gonopods in female paratype, ventral view; **B.** apical claw of gonopods in female paratype, dorsal view; **C.** apical claw of gonopods in female paratype, ventral view; **D.** gonopods in female paratype, lateral view; **E.** posterior segments and gonopods in male holotype, ventral view; **F.** male legs 15, holotype, dorsal view; **G.** femur of male legs 15, holotype, dorsal view. Arrowheads (B–D) refer to small subtriangular denticle.

Legs. Relative robust, tarsi fused on legs 1–13, well-defined on legs 14–15. All legs with moderately long curved claws; legs 1–13 with anterior and posterior accessory spurs, anterior accessory spurs moderately long and slender, forming a moderately small angle with the claw, posterior accessory spurs slightly more robust, forming a comparatively large angle with the claw; lacking accessory spurs of legs 14 and 15. From short to long setae sparsely scattered over the surface of coxa, trochanter, prefemur, femur and tibia of all legs, more setae on the tarsal surfaces; setae on the dorsal and ventral surfaces slightly longer than the anterior and posterior; some thicker setae arranged in one row on the ventral surfaces of tarsi of legs 1–13, with setae significantly reduced on legs 14 and 15. Legs 14 and 15 thicker than the anterior legs in both of the female and male, male legs 15 thicker and stronger than those of the female, with a shallow longitudinal groove on the dorsal surfaces of femur and tibia of the male legs 14 and 15 (Fig. 2F, G). Ta2 3.7–5.6 times longer than wide, Ta2 57.6%–79.2% length of Ta1 on legs 15 in female; Ta2 4.0–5.4 times longer than wide, Ta2 70.3%–83.5% length of Ta1 on legs 15 in male. Leg plectrotaxy given in Table 1 and Table 2.

Coxal pores. 3–5 in a row, 5-4-4-4(5) in female, 4-5-5(4)-4 and 3-4-4-3 in male; slightly oval or round, commonly round, coxal pore field set in a relatively shallow groove, the coxal pore-field fringe with a slight prominence and moderately long setae sparsely scattered over the surface.

Female. S15 anterior margin broader than posterior, generally an inverted trapezoid, pos­tero-medially concave. Moderately long setae sparsely scattered on S15 surface. Surface of the lateral sternal margin of genital segment well chitinised, posterior mar­gin of genital sternite deeply concave between condyles of gonopods, except for a small, median tongue-shaped bulge. Relatively long setae very sparsely scattered over ventral surface of the genital segment, slightly more setae on posterior part, especially along the posterior edge. Gonopods: first article fairly broad, bearing many moderately long setae; with 3+3 small coniform spurs, inner spur slightly smaller than the outer (Fig. 2A); second article with four or five long setae in the ventral, arranged in three irregular rows; three or four robust spines lying dorsally on the posterior part of the external margin, extending backwards and forming a moderately transparent protuberance; third article with three or four long setae in the ventral, three robust spines lying dorsally on the posterior part of the external margin, with a bidentate apical claw, with a very small subtriangular long pointed denticle on the ventral margin (Fig. 2B-D).

Male. S 15 posterior margin narrower than anterior, postero-medially straight, sparsely covered with long setae; sternite of genital segment evidently smaller than the female, usually sclerotised; posterior margin deeply concave between the gonopods, without medial bulge. Short to long setae equally scattered on the ventral surface of the genital segment. Gonopods short and small, appearing as small finger-like bulges, with two long setae, apically slightly sclerotised (Fig. 2E).

## Identification Keys

### Key to the Chinese species of the genus *Hessebius* Verhoeff, 1941

**Table:** 

1	Female gonopods having 3+3 spurs	*crassifemoralis* sp. n
–	Female gonopods having 2+2 spurs	2
2	Absent accessory spurs on legs 14^th^ and 15^th^	*ongispinipes* Ma, Pei & Zhu (Ma et al. 2009)
–	At least having an accessory spurs on legs 14^th^ and 15^th^	3
3	Dorsal sulci absent on male tibia of legs 14^th^ and 15^th^	4
–	Dorsal sulci present on male tibia of legs 14^th^ and 15^th^	6
4	Only posterior accessory spurs on legs 15^th^	*jangtseanus* Verhoeff (Verhoeff 1942)
–	Absent accessory spurs on legs 15^th^	5
5	Dorsal terminal thorn on 2^nd^ article of female gonopods extending backwards and forming a moderately transparent protuberance	*luculentus* Ma, Lu, Liu, Hou & Pei (Ma et al. 2018)
–	Dorsal terminal thorn on 2^nd^ article of female gonopods strongly extending backwards and a robust slightly curved spine at the end of the massive projection	*prospinosa* Qiao, Qin, Ma & Zhang (Qiao et al. 2019)
6	13–15 ocelli, apical claw of female gonopods simple and broad	*multiforaminis* Pei, Ma & Zhu (Pei et al. 2010)
–	7–10 ocelli, apical claw of female gonopods simple, slender and sharp, having small protuberance on ventral side	7
7	Two posterior ocelli is the largest, having posterior accessory spurs on legs 15^th^	*ruoergaiensis* Qiao, Qin, Ma, Su & Zhang (Qiao et al. 2018)
–	Posterior ocellus is the largest, absent accessory spurs on legs 15^th^	*luquensis* Qiao, Qin, Ma, Su & Zhang (Qiao et al. 2018)

## Discussion

The new species is morphologically close to *H.longispinipes* Ma, Pei & Zhu, 2009 from Xinjiang Uygur Autonomous Region, China SW, with which it shares antennae 18–23 articles, six to seven ocelli on each side arranged in three irregular rows, the posterior ocellus largest, 2+2 prosternal teeth, coxal pore formula of 3–5, dorsal sulci present on male tibia of legs 14^th^ and 15^th^ and legs 15^th^ lacking accessory spurs. However, they can be distinguished easily by the following characters. The new species absent dorsal posterior spine of tibia of legs 14 in contrast to present in H.longispinipes; DaC spine present on the legs 9–12 vs. absent in *H.longispinipes*; having 3+3 coniform-shaped spurs of female gonopods instead of 2+2 bullet-shaped spurs in *H.longispinipes*; apical claw of female gonopods bidentate instead of simple apical claw in *H.longispinipes*.

To assist in the identification of the Chinese species belonging to the genus *Hessebius*, an identification key is offered, emphasising characters that can be examined without high-magnification microscopy; moreover, these characters are specific to the taxa occurring in China.

## Supplementary Material

XML Treatment for Hessebius crassifemoralis

## Figures and Tables

**Table 1.** Leg plectrotaxy of *Hessebiuscrassifemoralis* sp. n., females (n = 8).

Legs	Ventral					Dorsal				
	C	Tr	P	F	Ti	C	Tr	P	F	Ti
1			p	am	m			p	a	a
2			p	am	m			p	ap	a
3			p	am	m			ap	ap	ap
4-5			p	am	am			ap	ap	ap
6			p	amp	am			ap	ap	ap
7-9			mp	amp	am			ap	ap	ap
10			mp	amp	am			amp	ap	ap
11			mp	amp	am			amp	ap	ap
12			mp	amp	am			amp	p	p
13		m	amp	amp	am	(a)		amp	p	p
14		m	amp	am	m	a		amp	p	
15		m	amp	am		a	(m)	amp		

Letters in brackets indicate variable spines.

**Table 2.** Leg plectrotaxy of *Hessebiuscrassifemoralis* sp. n., males (n = 4).

Legs	Ventral					Dorsal				
	C	Tr	P	F	Ti	C	Tr	P	F	Ti
1			p	a	am			p	a	a
2-3			p	am	am			p	ap	ap
4-6			p	am	am			p	ap	ap
7			p	am(p)	am			ap	ap	ap
8-10			mp	amp	am			ap	ap	ap
11-12			mp	amp	am			amp	ap	ap
13			mp	amp	(a)m			amp	p	p
14		m	amp	am	m			amp	p	
15		m	amp	am		a		amp	(p)	

Letters in brackets indicate variable spines.
